# Based on Network Pharmacology to Explore the Molecular Targets and Mechanisms of Gegen Qinlian Decoction for the Treatment of Ulcerative Colitis

**DOI:** 10.1155/2020/5217405

**Published:** 2020-11-24

**Authors:** Meiqi Wei, He Li, Qifang Li, Yi Qiao, Qun Ma, Ruining Xie, Rong Wang, Yuan Liu, Chao Wei, Bingbing Li, Canlei Zheng, Bing Sun, Bin Yu

**Affiliations:** ^1^Shandong University of Traditional Chinese Medicine, Jinan 250355, China; ^2^College of Integrated Chinese and Western Medicine, Jining Medical University, Jining 272067, China; ^3^Department of Traditional Chinese Medicine, Affiliated Hospital of Jining Medical University, Jining 272060, China; ^4^School of Public Health, Jining Medical University, Jining 272067, China; ^5^School of Basic Medicine, Jining Medical University, Jining 272067, China

## Abstract

**Background:**

Gegen Qinlian (GGQL) decoction is a common Chinese herbal compound for the treatment of ulcerative colitis (UC). In this study, we aimed to identify its molecular target and the mechanism involved in UC treatment by network pharmacology and molecular docking. *Material and Methods*. The active ingredients of *Puerariae*, *Scutellariae*, *Coptis*, and *Glycyrrhiza* were screened using the TCMSP platform with drug‐like properties (DL) ≥ 0.18 and oral availability (OB) ≥ 30%. To find the intersection genes and construct the TCM compound-disease regulatory network, the molecular targets were determined in the UniProt database and then compared with the UC disease differential genes with *P* value < 0.005 and ∣log2 (fold change) | >1 obtained in the GEO database. The intersection genes were subjected to protein-protein interaction (PPI) construction and Gene Ontology (GO) and Kyoto Encyclopedia of Genes and Genomes (KEGG) enrichment analysis. After screening the key active ingredients and target genes, the AutoDock software was used for molecular docking, and the best binding target was selected for molecular docking to verify the binding activity.

**Results:**

A total of 146 active compounds were screened, and quercetin, kaempferol, wogonin, and stigmasterol were identified as the active ingredients with the highest associated targets, and NOS2, PPARG, and MMP1 were the targets associated with the maximum number of active ingredients. Through topological analysis, 32 strongly associated proteins were found, of which EGFR, PPARG, ESR1, HSP90AA1, MYC, HSPA5, AR, AKT1, and RELA were predicted targets of the traditional Chinese medicine, and PPARG was also an intersection gene. It was speculated that these targets were the key to the use of GGQL in UC treatment. GO enrichment results showed significant enrichment of biological processes, such as oxygen levels, leukocyte migration, collagen metabolic processes, and nutritional coping. KEGG enrichment showed that genes were particularly enriched in the IL-17 signaling pathway, AGE-RAGE signaling pathway, toll-like receptor signaling pathway, tumor necrosis factor signaling pathway, transcriptional deregulation in cancer, and other pathways. Molecular docking results showed that key components in GGQL had good potential to bind to the target genes MMP3, IL1B, NOS2, HMOX1, PPARG, and PLAU.

**Conclusion:**

GGQL may play a role in the treatment of ulcerative colitis by anti-inflammation, antioxidation, and inhibition of cancer gene transcription.

## 1. Background

Ulcerative colitis (UC) is chronic nonspecific enteritis with typical clinical manifestations of abdominal pain, diarrhea, and mucopurulent bloody stools; it mostly manifests as superficial ulcers occurring in the rectum and sigmoid colon but can also spread to the proximal colon and even the entire colon, causing permanent fibrosis and tissue damage. Despite improvements in treatment prospects, the incidence of long-term colectomy has not decreased over the last 10 years, and achieving mucosal healing early may be strongly associated with a reduced risk of future colectomy [1].

Gegen Qinlian (GGQL) decoction is from *Treatise on Febrile and Miscellaneous Disease*, and the monarch drug *Pueraria* has antipyretic and antidiarrheal properties; meanwhile, the subject drugs *Scutellaria* and *Coptis* have a function to eliminate dampness and heat and *Glycyrrhiza* can replenish qi.

A meta-analysis including 2028 patients defined that GGQL could improve clinical symptoms and reduce an endoscopic severity index (UCEIS) and recurrence rate; further, it also led to fewer adverse events whether it was used alone or in combination with Western medicine [2]. According to spectral efficiency studies, its main components—puerarin, daidzin, and coptisine—act through a synergistic relationship, but the specific mechanism of action is not clear.

Network pharmacology combines pharmacology, bioinformatics, and several other sciences with system network analysis and explains the multicomponent and multitarget drug treatment mechanism from the direction of gene distribution, molecular function, and signaling pathways by constructing a network related to “disease-phenotype-gene-drug,” which is suitable for the study of traditional Chinese medicine (TCM) compounds.

## 2. Materials and Methods

### 2.1. Screening of Active Ingredients and Target Genes

The active ingredients of the GGQL herbs, including *Puerariae*, *Scutellariae*, *Coptis*, and *Glycyrrhiza*, were screened using the TCMSP platform (http://lsp.nwu.edu.cn/tcmsp.php) with drug‐like properties (DL) ≥ 0.18 and oral bioavailability (OB) ≥ 30% as conditions. The predicted targets of the screened compounds were acquired from the DrugBank (https://www.drugbank.ca/) database and verified literature. Meanwhile, the UniProt database (https://www.uniprot.org/) was used for comparison of target information and gene name standardization.

### 2.2. Acquisition of Differential Genes

The genetic samples—Series: GSE38713—of UC patients and healthy people were obtained from the GEO database (https://www.ncbi.nlm.nih.gov/geo/). The script was run in Strawberry Perl-5.30.2.1 (Perl) software, and the gene probe names were annotated as gene symbols and grouped. The “limma” package was installed in the Perl software, and the sample values were corrected and subjected to log2 (logFC) transformation. Samples with *P* value < 0.005 and ∣log2 (fold change) | >1 were screened and considered to have statistically significant differential genes. The gene volcano map of the samples was generated, and the top 20 genes with the most significant up- and downregulation were selected to draw the heat map.

### 2.3. Traditional Chinese Medicine Compound-Disease Regulatory Network

The Perl software was used to acquire intersection genes of the disease differential genes and the target genes of TCM, as well as the active ingredients of TCM. Subsequently, the TCM compound-disease regulatory network was generated using the Cytoscape software.

### 2.4. Protein-Protein Interaction (PPI) Network and Topological Analysis

The “bisogenet, cytoNAC” package was installed in the Cytoscape 3.8.0 software, and the intersection genes were entered, and the parameter “homo sapiens” was selected. Data for constructing the PPI network were sourced from six main experimental research databases: Database of Interacting Proteins, Biological General Repository for Interaction Datasets, Human Protein Reference Database, IntAct molecular interaction database, Molecular INTeraction Database, and Biomolecular Interaction Network Database. The method “input nodes and its neighbors” was selected to obtain the PPI network and perform topological analysis based on its network centrality.

### 2.5. GO and KEGG Enrichment Analysis

The R package including “colorspace,” “stringi,” and “ggplot2” was installed in software R 4.0.0, and a bioconductor package that includes “DOSE,” “clusterProfiler,” and “enrichplot” was used for GO and Kyoto Encyclopedia of Genes and Genomes (KEGG) enrichment analysis. The function “enrichGO” was used for GO enrichment analysis. The database was org. Hs .eg. db (DOI: 10.18129/http://b9.bioc.org.Hs.eg.db); the “enrichKEGG” function was applied for KEGG enrichment analysis, and the database was the KEGG database (https://www.kegg.jp/). As for the parameters of the two functions, species was set to “has,” and the filter values (i.e., *P* value and *q*-value) were set to 0.05. The first 20 enrichment results were visualized as a bar graph, and the KEGG regulatory network was generated by the Cytoscape 3.8.0 software.

### 2.6. Molecular Docking

The target genes involved in the first 10 pathways of the KEGG enrichment results were searched in the PDB database (https://www.rcsb.org), of which the 3D protein conformations with a crystal resolution of lower than 3 Å as determined by X crystal diffraction were acquired. The Mol2 format files of GGQL key active ingredients were downloaded from the TCMSP platform. The AutoDockTools 1.5.6 software was applied to process proteins as follows: separate proteins, add nonpolar hydrogen, calculate the Gasteiger charge, and assign the AD4 type, and set all the flexible bonds of small molecule ligands to be rotatable. According to the original ligand coordinates, the docking box was adjusted to include all protein structures. Meanwhile, the receptor protein was set to rigid docking, the genetic algorithm was selected, and the maximum number of evals was set as the medium. The docking results were obtained by running autogrid4 and autodock4, by which the binding energies were revealed. The partial diagram of molecular docking was then generated using the PyMol software.

## 3. Results

### 3.1. Screening of Active Ingredients and Target Genes

In the TCMSP database, GGQL contains 489 active ingredients in total, including 18 in *Puerariae*, 143 in *Scutellariae*, 48 in *Coptis*, and 280 in *Glycyrrhiza*. They were then screened with OB ≥ 30% and DL ≥ 0.18 as the conditions. Consequently, 146 active ingredients were obtained, including 4 in *Puerariae*, 36 in *Scutellariae*, 14 in *Coptis*, and 92 in *Glycyrrhiza* (Table 1). The targets were predicted by the DrugBank database and UniProt database. Eventually, 2660 targets—97 in *Puerariae*, 507 in *Scutellariae*, 287 in *Coptis*, and 1769 in *Glycyrrhiza*—were obtained.

### 3.2. Differential Gene Screening

**Figure 1:**
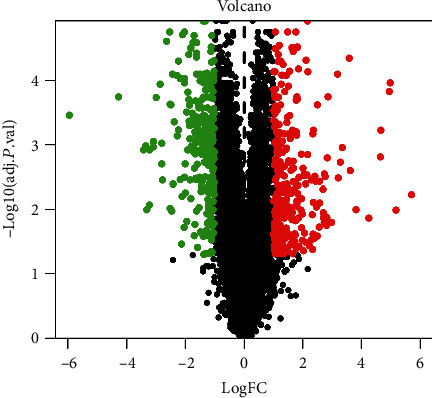
Gene volcano map shows the gene distribution in disease samples. Red and green represent upregulated genes (logFC > 0) and downregulated genes (logFC < 0), respectively, whereas black indicates no significant difference.

**Figure 2:**
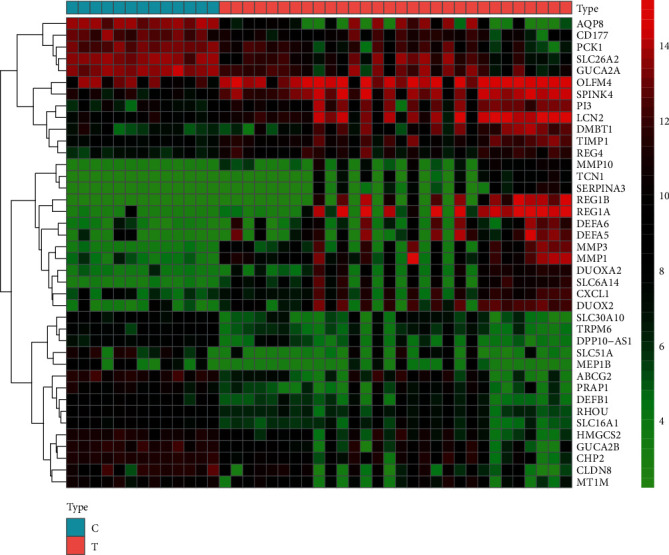
Gene heat map. In the gene heat map, red and green represent upregulated (logFC > 0) and downregulated (logFC < 0) genes in the sample, respectively, whereas black represents no significant difference. The first 13 samples were from healthy people, and the last 30 samples were from patients with ulcerative colitis.

By comparing 15 normal samples with 30 disease samples in the GEO database, a total of 21,653 differential genes were acquired, including 9198 upregulated genes and 12,455 downregulated genes. After screening with a *P* value < 0.005 and ∣log2 (fold change)  | >1, a total of 305 upregulated genes and 263 downregulated genes were obtained. As indicated by the gene volcano map (Figure 1), the differential genes in the disease samples display a normal distribution, with a larger number of significantly upregulated genes than significantly downregulated genes. The top 20 genes with the most significant upregulation and downregulation are presented in Figure 2 and Table 2.

### 3.3. Construction of the TCM Compound-Disease Regulatory Network

**Figure 3:**
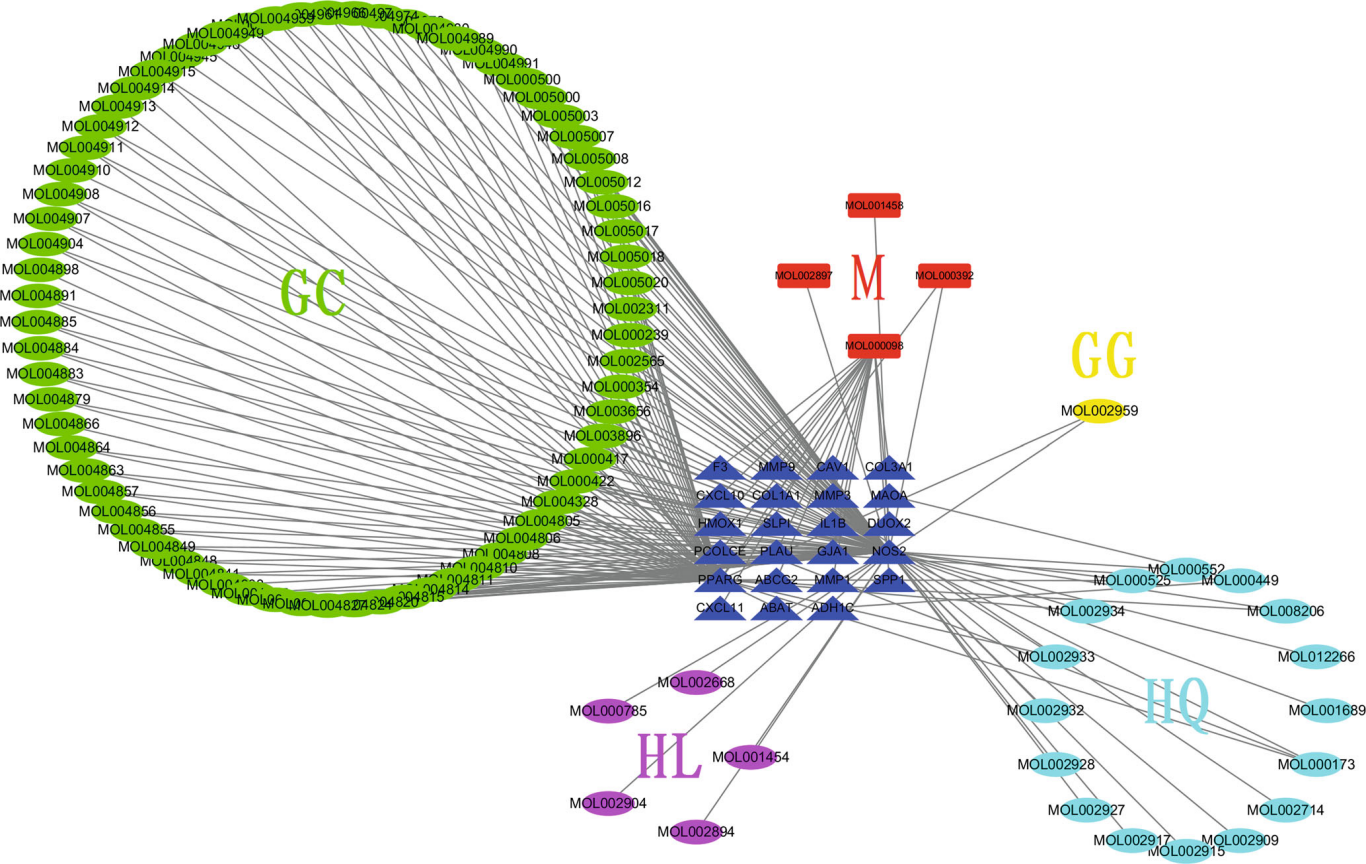
TCM compound-disease regulatory network. This network shows the targeted relationship between the active components of TCM and the intersection genes. Green GC represents *Glycyrrhiza*, yellow GG represents *Pueraria*, light blue HQ represents *Scutellaria*, purple HL represents *Coptis*, red M represents common components, and dark blue triangle represents intersection genes.

As listed in Table 3, there are 23 intersection genes (sorted by logFC). The targeting relationship between TCM active ingredients and intersection genes is presented by the TCM compound-disease regulatory network (Figure 3). Active ingredients of *Glycyrrhiza* and *Scutellariae* have the most amount of and related target genes, indicating that *Glycyrrhiza* and *Scutellariae* in GGQL are the most efficacious components. The active ingredients quercetin, kaempferol, wogonin, and stigmasterol are associated with 18, 5, 3, and 3 target genes, respectively. Therefore, they are classified as multitarget and multieffect compounds. The gene NOS2 is the gene associated with the highest number of active components, followed by PPARG and MMP1.

### 3.4. PPI Network and Topological Analysis

**Figure 4:**
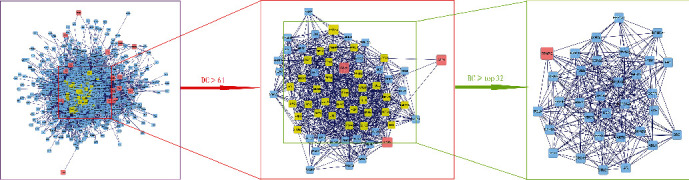
Topological analysis of the protein-protein interaction network. Herein, 830 protein nodes were obtained according to the intersection genes. After screening by DC > 61 for the first time, a total of 63 protein nodes were obtained, and the first 32 proteins were extracted according to BC for the second time.

In the PPI network, the degree centrality (DC) of a node is simply the number of edges it has. The higher the degree, the more central the node is. The betweenness centrality (BC) captures how much a given node is in between others. Specifically, it is the ratio of the number of the shortest paths passing through the node to the total number of the shortest paths in the network. DC and BC reflect the influence of the corresponding node in the entire network. They describe the topological centrality based on the connectivity and controllability of the network. The combination of DC and BC values has been confirmed to be effective for screening reliable important proteins [3]. As shown in Figure 4, 830 protein nodes and 9689 edges were obtained for intersection genes. After screening with DC > 61 and a BC range of 0–113.2, the first 32 proteins are shown in Table 4 (in descending order of degree), with a total of 273 edges. Among the 32 proteins, nine proteins are predicted targets of the active ingredients, with their corresponding genes being EGFR, PPARG, ESR1, HSP90AA1, MYC, HSPA5, AR, AKT1, and RELA.

### 3.5. Analysis of GO Function and KEGG Enrichment of Related Targets

**Figure 5:**
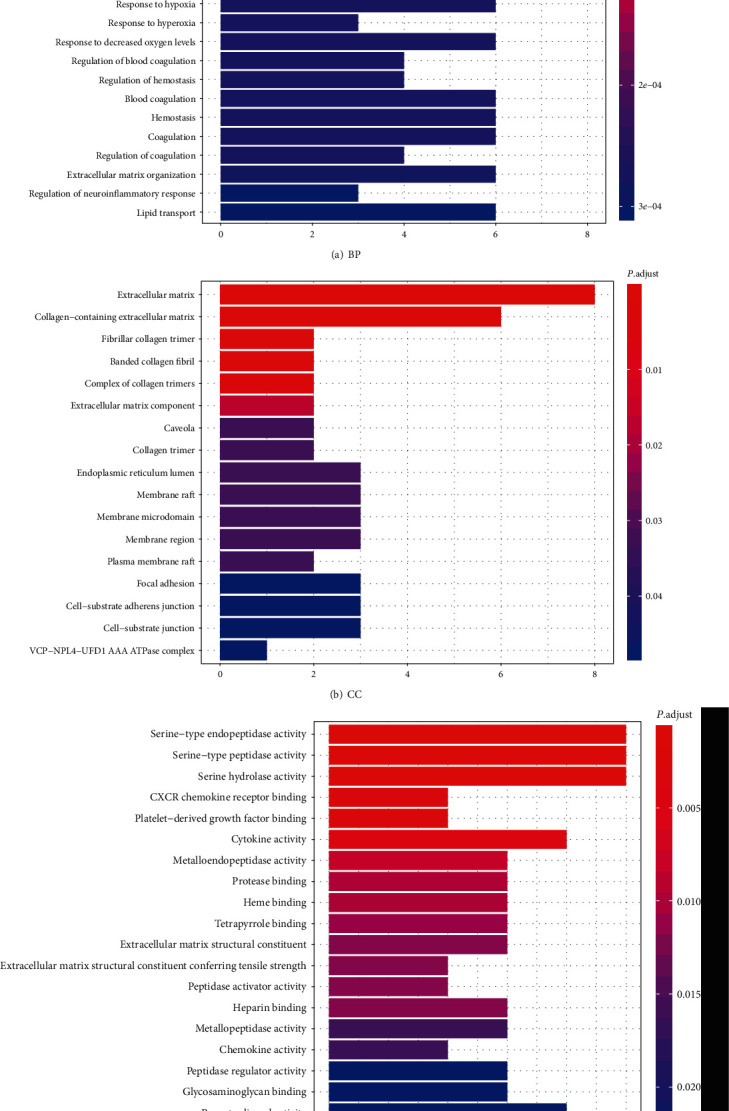
GO enrichment analysis of GGQL targets in treating UC. The horizontal axis of the BP, CC, and MF column represents the number of genes enriched in each item, and the color represents the enrichment significance based on the corrected *P* value.

**Figure 6:**
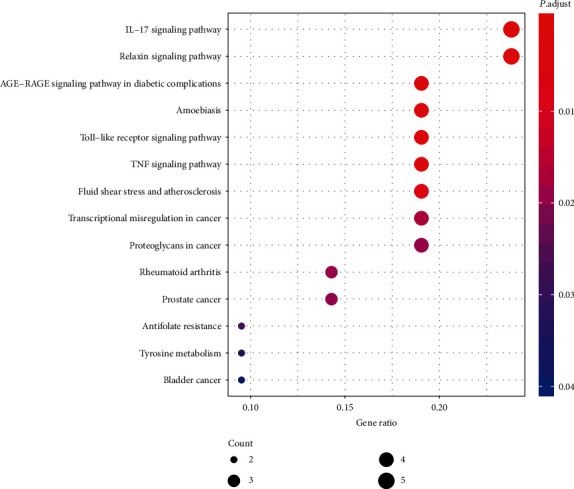
KEGG bubble. The horizontal axis of the KEGG bubble diagram represents the gene proportion enriched in each entry, and the vertical axis shows the enrichment degree according to the corrected *P* value.

**Figure 7:**
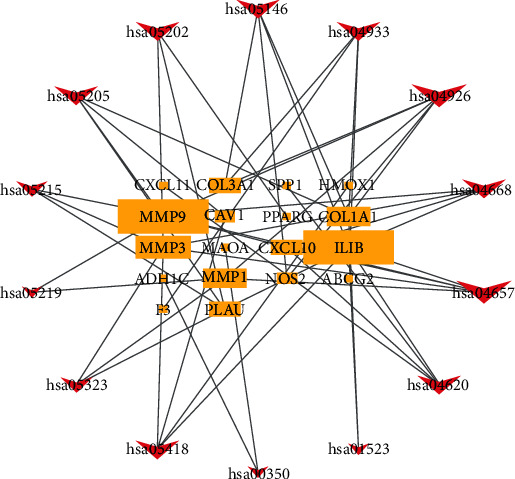
KEGG relational regulatory network. This network shows the relationship between the enriched 14 pathways and 18 genes, and the size of the graph shows the number of pathways or genes connected.

GO enrichment analysis illustrates gene function on three levels: biological process (BP), cellular component (CC), and molecular function (MF). BP mainly involves aspects of response to oxygen levels, leukocyte migration, collagen metabolic process, and response to nutrients. CC is mainly related to the extracellular matrix, collagen-containing extracellular matrix, fibrillar collagen trimer, and banded collagen fibril. MF is mainly involved in serine-type endopeptidase activity, serine-type peptidase activity, serine hydrolase activity, CXCR chemokine receptor binding, platelet-derived growth factor binding, and cytokine activity (Figure 5). According to KEGG enrichment results, the mechanism of GGQL in treating UC is mainly concentrated in the IL-17 signaling pathway, relaxin signaling pathway, AGE-RAGE signaling pathway in diabetic complications, toll-like receptor signaling pathway, TNF signaling pathway, fluid shear stress and atherosclerosis, transcriptional misregulation in cancer, proteoglycans in cancer, rheumatoid arthritis, and prostate cancer (Figure 6). Genes associated with the greatest number of pathways were IL1B, MMP9, and MMP3 (Figure 7 and Table 5).

### 3.6. Molecular Docking

**Figure 8:**
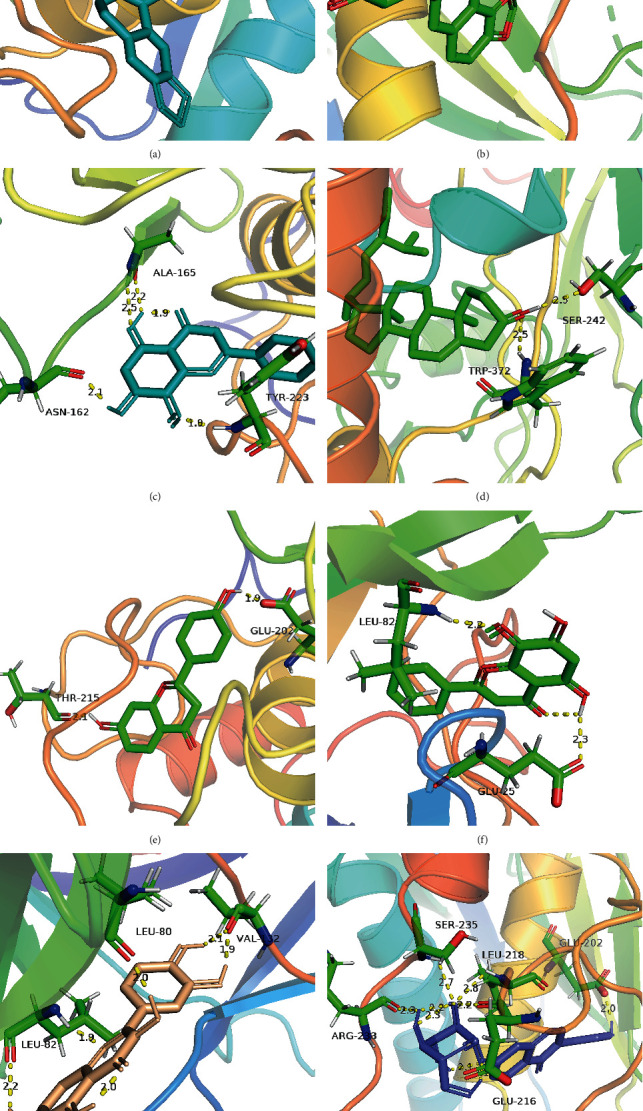
Partial diagram of molecular docking: (a) MMP3-berberine; (b) MMP3-coptisine; (c) MMP3-wogonin; (d) NOS2-stigmasterol; (e) MMP3-liquiritigenin; (f) IL1B-wogonin; (g) IL1B-quercetin; (h) MMP3-daidzin.

Molecular docking is a technique that mimics the interaction between small ligand molecules and receptor protein macromolecules, and the binding energy between the two counterparts can be calculated to predict their affinity. A binding energy lower than 0 indicates that the two molecules combine spontaneously and that smaller binding energies lead to more stable conformations. Most ingredients in GGQL can bind well with target genes, among which stigmasterol, coptisine, and berberine have the best binding properties (Table 6). The genes MMP3, IL1B, NOS2, HMOX1, PPARG, PLAU, and MMP1 can dock well with most active ingredients.  Figure 8 illustrates some local structures of molecular docking in detail.

## 4. Discussion

In this study, a network pharmacological analysis was conducted on the medicinal ingredients of the four TCMs (*Puerariae*, *Scutellariae*, *Coptis*, and *Glycyrrhiza*) in GGQL and UC disease targets. Quercetin, kaempferol, and wogonin were identified as the active ingredients associated with the most targets. The results of molecular docking also verified that they have good binding properties with most target genes. Quercetin is a common flavonoid compound in nature. It is considered the most effective reactive oxygen species (ROS) scavenger and inhibits the production of several proinflammatory factors, such as TNF-*α* and NO. The antioxidant and anti-inflammatory advantages of quercetin in UC treatment have been confirmed by various *in vivo* and *in vitro* experiments [4]. Quercetin plays an anticancer role by reducing the activity of kinase MEK1, downregulating the cascade reaction of Raf and MAPK, and inhibiting telomerase [5]. Kaempferol is also a natural flavonoid, and its efficacy as an anti-inflammatory, antioxidant, and anticancer agent has been reported in the treatment of a variety of diseases, such as diabetes, obesity, and cancer (e.g., skin, liver, and colon cancers) [6]. Wogonin is the compound with the highest content in *Scutellariae*; it is quickly converted into metabolites, such as baicalin, after entering the bloodstream. Baicalin has been confirmed to significantly inhibit TLR4-induced increase in NF-*κ*Bp65 levels, reduce the activity of enzymes, such as MPO and COX-2, lower the production of factors, such as THF-*α*, IL-1*β*, IL-12, and IFN-*γ*, and regulate the Th17/Treg cell balance [7, 8]. The molecular docking results indicate that stigmasterol, coptisine, and berberine have superior affinities to the target genes MMP3, IL1B, NOS2, HMOX1, PPARG, and PLAU, and they are the effective ingredients with potent anti-inflammatory and antioxidant effects. Stigmasterol has been shown to inhibit the innate immune response induced by lipopolysaccharide in a mouse model [9]. Berberine exerts local anti-inflammatory effects by blocking the IL-6/STAT3/NF-*κ*B signaling pathway. Meanwhile, it effectively enhances the expression of SIgA and lowers the expression of iNOS, MPO, and MDA [10].

A PPI topological analysis was performed for 23 intersection genes, revealing 32 strongly associated proteins, among which 9 proteins (EGFR, PPARG, ESR1, HSP90AA1, MYC, HSPA5, AR, AKT1, and RELA) are the predicted targets of TCMs. PPARG is also an intersection gene, and these 9 targets were speculated to be the key targets of GGQL in the treatment of UC.

UC is an inflammatory disease related to intestinal immune recognition disorders. The KEGG enrichment results indicate four inflammation-related pathways: IL-17 signaling pathway, AGE-RAGE signaling pathway in diabetic complications, toll-like receptor signaling pathway, and TNF signaling pathway. Meanwhile, further analysis revealed inflammation-related genes, such as IL1B, CXCL10, CXCL11, MMP9, MMP3, SPP1, NFKB1, IKBKG, and RELA. The first six genes are widely involved in IL-17, toll-like receptors, and TNF signaling pathways, while the latter three are participants in the NF-*κ*B pathway, particularly the gene RELA, which encodes NF-*κ*B p65. It is suggested by the results that both the treatment mechanism of GGQL and the pathogenesis of UC are related to inflammatory regulation. A meta-analysis showed that in allelic and dominant models, the genetic polymorphisms of IL-17A and IL-17F may increase the risk of UC occurrence. In addition, IL-17 levels in serum are significantly associated with the severity of UC [11]. Toll-like receptors (TLRs) are a group of transmembrane proteins widely distributed in immune cells, playing a key role in identifying invading pathogens and upregulating signals related to inflammatory cytokines and costimulatory molecules [12]. Tumor necrosis factor (TNF) not only is a potent proinflammatory mediator but also upregulates the production of ROS and RNS and exacerbates cell damage [13]. Anti-TNF therapy has been proven to quickly induce clinical and endoscopic remission in UC patients; however, its safety and related risks still require urgent attention [14]. NF-*κ*B induces cytokine expression and neutrophil aggregation. It is often regarded as a sign and the central pathway of inflammatory response, while being involved in cancer development through various pathways [15]. In the classical NF-*κ*B signaling pathway, TNF-*α* and IL-1 activate toll-like receptors (TLRs), followed by the activation of the I*κ*B kinase complex, which can phosphorylate I*κ*B*α* [15]. Advanced glycosylation end products (AGE) and IL-17 (highly expressed during UC activity) can also be used as mediators for NF-*κ*B pathway activation [16, 17]. In this study, it is confirmed that the action mechanism of GGQL is related to the four pathways of inflammatory response, and the regulation of these pathways is linked to the transcription of NF-*κ*B, indicating that GGQL plays its therapeutic role by inhibiting the inflammatory response mediated by the NF-*κ*B signaling pathway. It has also been confirmed by other studies that baicalin and berberine exert inflammatory inhibition effects by downregulating the expression of proinflammatory cytokines (i.e., IL-1*β*, TNF-*α*, ICAM-1, TLR2, and TLR4) and inhibiting the NF-*κ*Bp65 signal transduction pathway [18, 19].

The excessive ROS in the intestinal mucosa can trigger inflammatory responses by inducing redox-sensitive signaling pathways and transcription factors (e.g., NF-*κ*B, TNF-*α*, and AP-1). The inflammatory responses then lead to the generation of more ROS, forming a vicious circle between oxidative stress and inflammation [20]. The KEGG path map shows that there are three pathways related to oxidative stress: (1) relaxin signaling pathway, (2) fluid shear stress and atherosclerosis pathway that activates downstream second messengers of PI3K-AKT, thus inducing eNOS expression, and (3) AGE-RAGE signaling pathway in diabetic complications, in which the receptor RAGE activates NADPH oxidase 2, thus leading to excessive ROS production. PPARG, NOS2, and DUOX2 are the three key target genes closely related to oxidative stress. NOS2 and PPARG are the targets associated with most active ingredients. The results of molecular docking have also verified the effectiveness of the association. PPARG is an important gene for fatty acid metabolism and oxidation and insulin sensitization. It also inhibits the activation of NF-*κ*B to exert anti-inflammatory effects. PPAR-*γ* has been proven to be a key receptor for 5-ASA to exert anti-inflammatory and antioxidant effects [21]. Relevant studies have found that the expression of PPAR-*γ* messenger RNA in the colonic mucosa of UC patients is impaired, accompanied by enhanced expression of the corresponding inflammatory factors (e.g., NF-*κ*B). Partial PPAR-*γ* agonists may be a new target for UC treatment [22]. According to the results of differential genes, PPARG was downregulated. In GGQL, there are 67 compounds that have a regulatory effect on PPARG. NOS2 encodes the synthesis of inducible nitric oxide synthase (iNOS). The highly expressed iNOS in the inflamed mucosa plays a key role in oxidative stress-induced inflammation [23]. NOS activity in colon biopsies has been shown to be correlated with disease intensity [24]. DUOX2 participates in the regulation of hydrogen peroxide anabolism and mediates peroxidase activity on the mucosal surface. DUOXA2 is a partner of DUOX2. Significant upregulation was determined for DUOXA2, DUOX2, and NOS2, among the different genes. It implies that excessive production of ROS and RNS will bring oxidative damage to cellular components, including lipids, DNA, and proteins, thus causing damage to the mucosal barrier and leading to sustained inflammatory response [20]. In GGQL, 86 compounds, including quercetin and kaempferol, have been shown to have effects on NOS2 and DUOX2 by the TCM compound-disease regulatory network.

As suggested by the KEGG enrichment results, GGQL may prevent the occurrence of cancer in the following ways: transcriptional misregulation in cancer and proteoglycans in cancer. Persistent inflammatory response and oxidative stress state can promote the mutation of cancer genes. The abnormal expressions of NOS2, DUOX2/DUOXA2, ESR1, EGFR, MYC, and AKT1, which are being discussed in this study, have been confirmed to be related to cancer [25–28]. The expression of the protooncogene MYC is significantly upregulated in up to 70% of colorectal cancer (CRC) patients. The overexpression of its gene product, c-Myc, leads to the activation of downstream genes, DNA synthesis, cell proliferation, and chromosomal aberrations. These mechanisms ultimately lead to genomic instability and chemical resistance [29]. Encoded by the EGFR gene, EGFR is a transmembrane glycoprotein belonging to the ErbB family; it is reportedly excessively expressed in 85% of NSCLC cells and is associated with a poor prognosis. By regulating downstream signaling pathways, mainly PI3K/Akt and MAPK pathways, the activated EGFR leads to receptor dimerization and tyrosine autophosphorylation, which could result in aberrant proliferation in certain cells, such as NSCLC cells. MYC is a protooncogene that encodes transcription factors involved in basic cellular pathophysiological processes. Activation of MYC causes abnormal cell proliferation, regression, and redifferentiation of cancer cells and susceptibility to aurora kinase inhibition in SCLC cells [30]. Proteoglycans are the main components of the extracellular matrix. They participate in matrix remodeling in tumor cell growth and the formation of stromal vessels, thus affecting the response of tumor cells and tissues and regulating the cancer phenotype by influencing the signals within cancer cells [31].

Many other scholars' studies are consistent with our findings. Xu et al. found that GGQL decoction could increase the SOD activity and decrease the MDA and iNOS activities along with the reduction in TNF-*α* and IL-1*β* levels to take effect on the treatment of UC rats [18]. Li et al. revealed that GGQL could reduce the TLR4 expression and NF-*κ*B activation along with several inflammatory cytokines such as TNF-*α*, IL-6, IL-1*β*, and IL-4 and NO [32].

This study shows that the mechanism of GGQL in UC treatment is related to its anti-inflammatory and antioxidative properties and inhibition of oncogene transcription. Meanwhile, the action targets are related to NOS2, PPARG, and MMP1. This study has not demonstrated the relationship between the mechanism of GGQL treatment and signaling pathways of relaxin, amoebiasis, and prostate cancer. Relevant studies on this are based on existing database information and lack experimental verification. Therefore, it is worthy of subsequent experimental analysis to confirm the reliability of the results.

## Figures and Tables

**Table 1:** The total available compounds of GGQL.

Drug	ID	Compound	OB (%)	DL
Pueraria	MOL000392	Formononetin	69.67	0.21
Pueraria	MOL000358	Beta-sitosterol	36.91	0.75
Pueraria	MOL002959	3′-Methoxydaidzein	48.57	0.24
Pueraria	MOL003629	Daidzein-4,7-diglucoside	47.27	0.67
Scutellaria	MOL001689	Acacetin	34.97	0.24
Scutellaria	MOL000173	Wogonin	30.68	0.23
Scutellaria	MOL000228	(2R)-7-Hydroxy-5-methoxy-2-phenylchroman-4-one	55.23	0.2
Scutellaria	MOL002714	Baicalein	33.52	0.21
Scutellaria	MOL002908	5,8,2′-Trihydroxy-7-methoxyflavone	37.01	0.27
Scutellaria	MOL002909	5,7,2,5-Tetrahydroxy-8,6-dimethoxyflavone	33.82	0.45
Scutellaria	MOL002910	Carthamidin	41.15	0.24
Scutellaria	MOL002911	2,6,2′,4′-Tetrahydroxy-6′-methoxychaleone	69.04	0.22
Scutellaria	MOL002913	Dihydrobaicalin_qt	40.04	0.21
Scutellaria	MOL002914	Eriodyctiol (flavanone)	41.35	0.24
Scutellaria	MOL002915	Salvigenin	49.07	0.33
Scutellaria	MOL002917	5,2′,6′-Trihydroxy-7,8-dimethoxyflavone	45.05	0.33
Scutellaria	MOL002925	5,7,2′,6′-Tetrahydroxyflavone	37.01	0.24
Scutellaria	MOL002926	Dihydrooroxylin A	38.72	0.23
Scutellaria	MOL002927	Skullcapflavone II	69.51	0.44
Scutellaria	MOL002928	Oroxylin A	41.37	0.23
Scutellaria	MOL002932	Panicolin	76.26	0.29
Scutellaria	MOL002933	5,7,4′-Trihydroxy-8-methoxyflavone	36.56	0.27
Scutellaria	MOL002934	Neobaicalein	104.34	0.44
Scutellaria	MOL002937	Dihydrooroxylin	66.06	0.23
Scutellaria	MOL000358	Beta-sitosterol	36.91	0.75
Scutellaria	MOL000359	Sitosterol	36.91	0.75
Scutellaria	MOL000525	Norwogonin	39.4	0.21
Scutellaria	MOL000552	5,2′-Dihydroxy-6,7,8-trimethoxyflavone	31.71	0.35
Scutellaria	MOL000073	Ent-epicatechin	48.96	0.24
Scutellaria	MOL000449	Stigmasterol	43.83	0.76
Scutellaria	MOL001458	Coptisine	30.67	0.86
Scutellaria	MOL001490	Bis[(2S)-2-ethylhexyl] benzene-1,2-dicarboxylate	43.59	0.35
Scutellaria	MOL001506	Supraene	33.55	0.42
Scutellaria	MOL002879	Diop	43.59	0.39
Scutellaria	MOL002897	Epiberberine	43.09	0.78
Scutellaria	MOL008206	Moslosooflavone	44.09	0.25
Scutellaria	MOL010415	11,13-Eicosadienoic acid, methyl ester	39.28	0.23
Scutellaria	MOL012245	5,7,4′-Trihydroxy-6-methoxyflavanone	36.63	0.27
Scutellaria	MOL012246	5,7,4′-Trihydroxy-8-methoxyflavanone	74.24	0.26
Scutellaria	MOL012266	Rivularin	37.94	0.37
Coptis	MOL001454	Berberine	36.86	0.78
Coptis	MOL013352	Obacunone	43.29	0.77
Coptis	MOL002894	Berberrubine	35.74	0.73
Coptis	MOL002897	Epiberberine	43.09	0.78
Coptis	MOL002903	(R)-Canadine	55.37	0.77
Coptis	MOL002904	Berlambine	36.68	0.82
Coptis	MOL002907	Corchoroside A_qt	104.95	0.78
Coptis	MOL000622	Magnograndiolide	63.71	0.19
Coptis	MOL000762	Palmidin A	35.36	0.65
Coptis	MOL000785	Palmatine	64.6	0.65
Coptis	MOL000098	Quercetin	46.43	0.28
Coptis	MOL001458	Coptisine	30.67	0.86
Coptis	MOL002668	Worenine	45.83	0.87
Coptis	MOL008647	Moupinamide	86.71	0.26
Glycyrrhiza	MOL001484	Inermine	75.18	0.54
Glycyrrhiza	MOL001792	DFV	32.76	0.18
Glycyrrhiza	MOL000211	Mairin	55.38	0.78
Glycyrrhiza	MOL002311	Glycyrol	90.78	0.67
Glycyrrhiza	MOL000239	Jaranol	50.83	0.29
Glycyrrhiza	MOL002565	Medicarpin	49.22	0.34
Glycyrrhiza	MOL000354	Isorhamnetin	49.6	0.31
Glycyrrhiza	MOL000359	Sitosterol	36.91	0.75
Glycyrrhiza	MOL003656	Lupiwighteone	51.64	0.37
Glycyrrhiza	MOL003896	7-Methoxy-2-methyl isoflavone	42.56	0.2
Glycyrrhiza	MOL000392	Formononetin	69.67	0.21
Glycyrrhiza	MOL000417	Calycosin	47.75	0.24
Glycyrrhiza	MOL000422	Kaempferol	41.88	0.24
Glycyrrhiza	MOL004328	Naringenin	59.29	0.21
Glycyrrhiza	MOL004805	(2S)-2-[4-Hydroxy-3-(3-methylbut-2-enyl)phenyl]-8,8-dimethyl-2,3-dihydropyrano[2,3-f]chromen-4-one	31.79	0.72
Glycyrrhiza	MOL004806	Euchrenone	30.29	0.57
Glycyrrhiza	MOL004808	Glyasperin B	65.22	0.44
Glycyrrhiza	MOL004810	Glyasperin F	75.84	0.54
Glycyrrhiza	MOL004811	Glyasperin C	45.56	0.4
Glycyrrhiza	MOL004814	Isotrifoliol	31.94	0.42
Glycyrrhiza	MOL004815	(E)-1-(2,4-Dihydroxyphenyl)-3-(2,2-dimethylchromen-6-yl)prop-2-en-1-one	39.62	0.35
Glycyrrhiza	MOL004820	Kanzonol W	50.48	0.52
Glycyrrhiza	MOL004824	(2S)-6-(2,4-Dihydroxyphenyl)-2-(2-hydroxypropan-2-yl)-4-methoxy-2,3-dihydrofuro[3,2-g]chromen-7-one	60.25	0.63
Glycyrrhiza	MOL004827	Semilicoisoflavone B	48.78	0.55
Glycyrrhiza	MOL004828	Glepidotin A	44.72	0.35
Glycyrrhiza	MOL004829	Glepidotin B	64.46	0.34
Glycyrrhiza	MOL004833	Phaseolinisoflavan	32.01	0.45
Glycyrrhiza	MOL004835	Glypallichalcone	61.6	0.19
Glycyrrhiza	MOL004838	8-(6-Hydroxy-2-benzofuranyl)-2,2-dimethyl-5-chromenol	58.44	0.38
Glycyrrhiza	MOL004841	Licochalcone B	76.76	0.19
Glycyrrhiza	MOL004848	Licochalcone G	49.25	0.32
Glycyrrhiza	MOL004849	3-(2,4-Dihydroxyphenyl)-8-(1,1-dimethylprop-2-enyl)-7-hydroxy-5-methoxy-coumarin	59.62	0.43
Glycyrrhiza	MOL004855	Licoricone	63.58	0.47
Glycyrrhiza	MOL004856	Gancaonin A	51.08	0.4
Glycyrrhiza	MOL004857	Gancaonin B	48.79	0.45
Glycyrrhiza	MOL004860	Licorice glycoside E	32.89	0.27
Glycyrrhiza	MOL004863	3-(3,4-Dihydroxyphenyl)-5,7-dihydroxy-8-(3-methylbut-2-enyl)chromone	66.37	0.41
Glycyrrhiza	MOL004864	5,7-Dihydroxy-3-(4-methoxyphenyl)-8-(3-methylbut-2-enyl)chromone	30.49	0.41
Glycyrrhiza	MOL004866	2-(3,4-Dihydroxyphenyl)-5,7-dihydroxy-6-(3-methylbut-2-enyl)chromone	44.15	0.41
Glycyrrhiza	MOL004879	Glycyrin	52.61	0.47
Glycyrrhiza	MOL004882	Licocoumarone	33.21	0.36
Glycyrrhiza	MOL004883	Licoisoflavone	41.61	0.42
Glycyrrhiza	MOL004884	Licoisoflavone B	38.93	0.55
Glycyrrhiza	MOL004885	Licoisoflavanone	52.47	0.54
Glycyrrhiza	MOL004891	Shinpterocarpin	80.3	0.73
Glycyrrhiza	MOL004898	(E)-3-[3,4-Dihydroxy-5-(3-methylbut-2-enyl)phenyl]-1-(2,4-dihydroxyphenyl)prop-2-en-1-one	46.27	0.31
Glycyrrhiza	MOL004903	Liquiritin	65.69	0.74
Glycyrrhiza	MOL004904	Licopyranocoumarin	80.36	0.65
Glycyrrhiza	MOL004905	3,22-Dihydroxy-11-oxo-delta(12)-oleanene-27-alpha-methoxycarbonyl-29-oic acid	34.32	0.55
Glycyrrhiza	MOL004907	Glyzaglabrin	61.07	0.35
Glycyrrhiza	MOL004908	Glabridin	53.25	0.47
Glycyrrhiza	MOL004910	Glabranin	52.9	0.31
Glycyrrhiza	MOL004911	Glabrene	46.27	0.44
Glycyrrhiza	MOL004912	Glabrone	52.51	0.5
Glycyrrhiza	MOL004913	1,3-Dihydroxy-9-methoxy-6-benzofurano[3,2-c]chromenone	48.14	0.43
Glycyrrhiza	MOL004914	1,3-Dihydroxy-8,9-dimethoxy-6-benzofurano[3,2-c]chromenone	62.9	0.53
Glycyrrhiza	MOL004915	Eurycarpin A	43.28	0.37
Glycyrrhiza	MOL004917	Glycyroside	37.25	0.79
Glycyrrhiza	MOL004924	(-)-Medicocarpin	40.99	0.95
Glycyrrhiza	MOL004935	Sigmoidin B	34.88	0.41
Glycyrrhiza	MOL004941	(2R)-7-Hydroxy-2-(4-hydroxyphenyl)chroman-4-one	71.12	0.18
Glycyrrhiza	MOL004945	(2S)-7-Hydroxy-2-(4-hydroxyphenyl)-8-(3-methylbut-2-enyl)chroman-4-one	36.57	0.32
Glycyrrhiza	MOL004948	Isoglycyrol	44.7	0.84
Glycyrrhiza	MOL004949	Isolicoflavonol	45.17	0.42
Glycyrrhiza	MOL004957	HMO	38.37	0.21
Glycyrrhiza	MOL004959	1-Methoxyphaseollidin	69.98	0.64
Glycyrrhiza	MOL004961	Quercetin der.	46.45	0.33
Glycyrrhiza	MOL004966	3′-Hydroxy-4′-O-methylglabridin	43.71	0.57
Glycyrrhiza	MOL000497	Licochalcone A	40.79	0.29
Glycyrrhiza	MOL004974	3′-Methoxyglabridin	46.16	0.57
Glycyrrhiza	MOL004978	2-[(3R)-8,8-Dimethyl-3,4-dihydro-2H-pyrano[6,5-f]chromen-3-yl]-5-methoxyphenol	36.21	0.52
Glycyrrhiza	MOL004980	Inflacoumarin A	39.71	0.33
Glycyrrhiza	MOL004985	Icos-5-enoic acid	30.7	0.2
Glycyrrhiza	MOL004988	Kanzonol F	32.47	0.89
Glycyrrhiza	MOL004989	6-Prenylated eriodictyol	39.22	0.41
Glycyrrhiza	MOL004990	7,2′,4′-Trihydroxy-5-methoxy-3-arylcoumarin	83.71	0.27
Glycyrrhiza	MOL004991	7-Acetoxy-2-methylisoflavone	38.92	0.26
Glycyrrhiza	MOL004993	8-Prenylated eriodictyol	53.79	0.4
Glycyrrhiza	MOL004996	Gadelaidic acid	30.7	0.2
Glycyrrhiza	MOL000500	Vestitol	74.66	0.21
Glycyrrhiza	MOL005000	Gancaonin G	60.44	0.39
Glycyrrhiza	MOL005001	Gancaonin H	50.1	0.78
Glycyrrhiza	MOL005003	Licoagrocarpin	58.81	0.58
Glycyrrhiza	MOL005007	Glyasperin M	72.67	0.59
Glycyrrhiza	MOL005008	Glycyrrhiza flavonol A	41.28	0.6
Glycyrrhiza	MOL005012	Licoagroisoflavone	57.28	0.49
Glycyrrhiza	MOL005013	18*α*-Hydroxyglycyrrhetic acid	41.16	0.71
Glycyrrhiza	MOL005016	Odoratin	49.95	0.3
Glycyrrhiza	MOL005017	Phaseol	78.77	0.58
Glycyrrhiza	MOL005018	Xambioona	54.85	0.87
Glycyrrhiza	MOL005020	Dehydroglyasperin C	53.82	0.37
Glycyrrhiza	MOL000098	Quercetin	46.43	0.28

**Table 2:** The first 20 genes that are upregulated and downregulated.

Gene names	LogFC	*P* value	Regulation direction
REG1A	5.701613746	0.00052702	Up
REG1B	5.1898523	0.001131623	Up
SLC6A14	4.991420548	8.72*E* − 07	Up
MMP1	4.963627094	1.48*E* − 06	Up
MMP3	4.668142374	1.70*E* − 05	Up
DUOX2	4.65206741	7.48*E* − 05	Up
DEFA5	4.261312381	0.001683584	Up
DEFA6	3.819380723	0.001118108	Up
DUOXA2	3.628039842	0.000154156	Up
SPINK4	3.590142516	1.56*E* − 07	Up
LCN2	3.354560231	4.42*E* − 05	Up
PI3	3.28851345	0.000101703	Up
MMP10	3.213180338	0.000222541	Up
REG4	3.19089349	4.90*E* − 07	Up
DMBT1	2.987003705	0.002100636	Up
TIMP1	2.864973614	2.15*E* − 06	Up
OLFM4	2.838270116	7.87*E* − 05	Up
CXCL1	2.822789462	0.001668369	Up
SERPINA3	2.811244589	0.002463382	Up
TCN1	2.768089208	0.001564425	Up
AQP8	−5.946044173	6.72*E* − 06	Down
ABCG2	−4.278991642	2.21*E* − 06	Down
PCK1	−3.426343127	5.08*E* − 05	Down
GUCA2B	−3.353230111	4.09*E* − 05	Down
SLC51A	−3.319192058	0.001123545	Down
PRAP1	−3.226028948	5.10*E* − 05	Down
CLDN8	−3.223765137	0.000875494	Down
SLC26A2	−3.096598258	4.32*E* − 05	Down
HMGCS2	−3.089377316	3.28*E* − 05	Down
SLC30A10	−2.994061586	2.34*E* − 06	Down
DEFB1	−2.862625712	9.37*E* − 07	Down
CHP2	−2.806425883	0.000109215	Down
CD177	−2.802954854	3.61*E* − 05	Down
GUCA2A	−2.776457399	0.000245692	Down
TRPM6	−2.636295344	3.93*E* − 08	Down
RHOU	−2.531238896	1.72*E* − 08	Down
DPP10-AS1	−2.521195069	3.47*E* − 06	Down
MT1M	−2.518431028	0.001039377	Down
SLC16A1	−2.492300121	3.72*E* − 06	Down
MEP1B	−2.462876667	0.001188996	Down

**Table 3:** The 23 intersection genes sorted by logFC.

Intersection gene	LogFC	*P* value
MMP1	4.963627094	1.48*E* − 06
MMP3	4.668142374	1.69963*E* − 05
DUOX2	4.65206741	7.47983*E* − 05
PLAU	2.323571552	0.0014514
IL1B	2.234056	0.006640247
NOS2	2.061560817	0.003623843
GJA1	2.011116812	0.000136087
MMP9	1.867276075	0.006997334
CXCL10	1.78792717	0.00738838
CXCL11	1.728830977	0.005420693
COL3A1	1.308597667	0.001234088
PCOLCE	1.293546472	0.006822444
CAV1	1.2259564	0.00238233
SLPI	1.163710008	0.002878356
F3	1.139466187	0.001216101
SPP1	1.041938337	0.004669085
COL1A1	1.022467924	0.002151629
MAOA	−1.077796648	0.000138579
ABAT	−1.190239356	9.18*E* − 06
HMOX1	−1.369875348	8.05*E* − 06
PPARG	−1.377777361	6.87*E* − 06
ADH1C	−2.038856081	0.002719353
ABCG2	−4.278991642	2.21*E* − 06

**Table 4:** Topological analysis results by degree—the first 32 proteins.

Gene names	Annotation	Degree	Betweenness
NTRK1	Neurotrophic receptor tyrosine kinase 1	239	79.2792394
EGFR	Epidermal growth factor receptor	170	57.27745249
FN1	Fibronectin	169	36.34401789
UBC	Ubiquitin C	157	50.80248406
PPARG	Peroxisome proliferator-activated receptor gamma	153	37.99948007
ESR1	Estrogen receptor 1	150	113.2071674
HSP90AA1	Heat shock protein 90 alpha family class A member 1	132	103.9892463
VCP	Valosin-containing protein	121	53.86564087
YWHAZ	Tyrosine 3-monooxygenase/tryptophan 5-monooxygenase activation protein zeta	119	64.36717285
MYC	v-myc avian myelocytomatosis viral oncogene homolog	112	77.51063594
HSPA5	Heat shock protein family A (Hsp70) member 5	108	34.49237132
NPM1	Nucleophosmin	106	63.99301971
HSP90AB1	Heat shock protein 90 alpha family class B member 1	104	52.53742327
COPS5	COP9 signalosome subunit 5	102	84.17298609
EP300	E1A-binding protein p300	98	59.70375531
SRC	SRC protooncogene, nonreceptor tyrosine kinase	94	52.01196867
AR	Androgen receptor	93	39.60526919
BRCA1	BRCA1, DNA repair associated	92	37.41150073
MDM2	MDM2 protooncogene	88	56.58152297
AKT1	AKT serine/threonine kinase 1	87	57.04410728
CTNNB1	Catenin beta 1	85	44.34296823
HSPA4	Heat shock protein family A (Hsp70) member 4	83	59.0919423
EEF1A1	Eukaryotic translation elongation factor 1 alpha 1	81	44.07432309
SMAD2	SMAD family member 2	78	38.64577814
RELA	RELA protooncogene, NF-*κ*B subunit	77	40.34807667
NFKB1	Nuclear factor kappa B subunit 1	75	41.54441528
TUBB	Tubulin beta class I	72	85.09134103
IKBKG	Inhibitor of nuclear factor kappa B kinase subunit gamma	69	40.75351178
HNRNPA1	Heterogeneous nuclear ribonucleoprotein A1	67	45.38051383
PRKDC	Protein kinase, DNA-activated, catalytic polypeptide	66	45.37484081
ABL1	ABL protooncogene 1, nonreceptor tyrosine kinase	65	34.38427075
STUB1	STIP1 homology and U-box-containing protein 1	64	41.4704054

**Table 5:** The enrichment pathways corresponding to intersection genes.

Term	Description	Count	Gene ID
hsa04657	IL-17 signaling pathway	5	MMP1/MMP3/MMP9/IL1B/CXCL10
hsa04926	Relaxin signaling pathway	5	NOS2/MMP1/MMP9/COL1A1/COL3A1
hsa04933	AGE-RAGE signaling pathway in diabetic complications	4	F3/IL1B/COL1A1/COL3A1
hsa05146	Amoebiasis	4	NOS2/IL1B/COL1A1/COL3A1
hsa04620	Toll-like receptor signaling pathway	4	IL1B/CXCL11/CXCL10/SPP1
hsa04668	TNF signaling pathway	4	MMP3/MMP9/IL1B/CXCL10
hsa05418	Fluid shear stress and atherosclerosis	4	HMOX1/MMP9/CAV1/IL1B
hsa05202	Transcriptional misregulation in cancer	4	PPARG/MMP3/PLAU/MMP9
hsa05323	Rheumatoid arthritis	3	MMP1/MMP3/IL1B
hsa05205	Proteoglycans in cancer	4	PLAU/MMP9/CAV1/COL1A1
hsa05215	Prostate cancer	3	MMP3/PLAU/MMP9
hsa01523	Antifolate resistance	2	IL1B/ABCG2
hsa00350	Tyrosine metabolism	2	ADH1C/MAOA
hsa05219	Bladder cancer	2	MMP1/MMP9

**Table 6:** Binding energies of GGQL's key components to the target gene molecules.

Key components	Binding energies (kcal/mol)											
	MMP3	IL1B	NOS2	HMOX1	PPARG	PLAU	MMP1	MMP9	COCL10	COL1A1	COL3A1	SPP1
Stigmasterol	−10.3	−8.78	−8.75	−8.04	−7.64	−7.25	−7.22	−6.94	−5.94	−6.32	−6.06	−6.9
Coptisine	−9.27	−7.97	−6.97	−6.27	−6.79	−6.59	−6.58	−6.73	−4.57	−6.31	−6.46	−6.16
Berberine	−9.81	−7.71	−6.92	−6.36	−6.57	−7.29	−6.81	−6.43	−4.84	−5.63	−5.89	−6.01
Liquiritigenin	−8.63	−7.95	−6.39	−6.04	−7.06	−7.41	−5.61	−7.58	−5.88	−5.89	−5.77	−4.31
Quercetin	−7.73	−8.45	−5.04	−5.2	−5.55	−7.17	−5.44	−5.05	−4.67	−3.92	−4.26	−5.09
Kaempferol	−8.37	−7.89	−5.82	−6.59	−6.2	−6.7	−4.79	−5.09	−5.38	−4.03	−5.37	−5.16
Wogonin	−8.96	−7.42	−5.98	−6.23	−5.91	−6.89	−4.74	−5.72	−4.48	−4.9	−5.22	−5.34
Baicalein	−8.41	−7.23	−6.59	−5.27	−6.67	−7.28	−5.65	−7.12	−5.19	−4.62	−6.16	−5.57
Puerarin	−6.46	−7.19	−4.95	−4.77	−4.93	−4.43	−4.07	−3.37	−2.73	−3.28	−4.39	−3.92
Daidzin	−8.7	−6.76	−6.33	−4.15	−4.52	−6.01	−4.27	−6.65	−4.07	−3.4	−4.56	−4.67
Epiberberine	−8.54	−7.38	−7.31	−6.19	−6.24	−6.19	−6.25	−6.83	−4.62	−6.12	−5.82	−5.53
Jatrorrhizine	−7.54	−7.46	−5.88	−5.01	−6.02	−5.44	−5.17	−5.92	−4.36	−5.1	−4.45	−4.98
Baicalin	−5.76	−6.69	−4.69	−3.49	−4.52	−4.22	−4.52	−2.41	−2.98	−2.98	−4.29	−3.82
Palmatine	−7.75	−6.8	−5.89	−5.81	−4.97	−5.76	−6.36	−5.55	−3.9	−5.23	−5.68	−5.3
Wogonoside	−7.68	−8.5	−5.99	−4.83	−5.22	−6.5	−4.73	−3.13	−5.36	−3.38	−4.49	−5.53

## Data Availability

The data that support the findings of this study are available from the corresponding author upon request.
